# A rare polyoxometalate cluster [NiW_12_O_44_]^14−^ based solid as a pre-catalyst for efficient and long-term oxygen evolution†

**DOI:** 10.1039/d2na00646d

**Published:** 2022-10-13

**Authors:** Parul Sood, Arti Joshi, Monika Singh

**Affiliations:** Institute of Nano Science and Technology Knowledge City, Sector-81 Mohali Punjab India monika@inst.ac.in

## Abstract

Polyoxometalates (POMs) are an eminent class of metal oxide anionic clusters of early transition metals with huge structural diversity. Herein, a [NiW_12_O_44_]^14−^ cluster based solid, (C_5_H_7_N_2_)_6_[NiW_12_O_44_], has been reported (PS-78). The [NiW_12_O_44_]^14−^ cluster bridges the missing gap of 1 : 12 hetero-POMs of Keggin and Silverton together with a coordination number of 8 of the central heteroatom (Ni). Furthermore PS-78 has been explored as an efficient and highly sustained oxygen evolution pre-catalyst in alkaline medium with an overpotential of 347 mV to attain a current density of 10 mA cm^−2^ and long-term stability up to 96 hours. Furthermore, mechanistic investigation showed that *in situ* generated NiO and WO_*x*_ (*x* = 1, 2) species act as active species for the oxygen evolution reaction. This study will open up new avenues for exploring POMs' new topologies and the potential of POMs as effective pre-catalysts in electrocatalytic applications.

## Abstract 2

A rare POM cluster [NiW_12_O_44_]^14−^ based solid was explored for OER. It acts as an efficient and highly sustained pre-catalyst in alkaline medium with an overpotential of 347 mV to achieve a current density of 10 mA cm^−2^ and stability up to 96 h.

## Graphical abstract

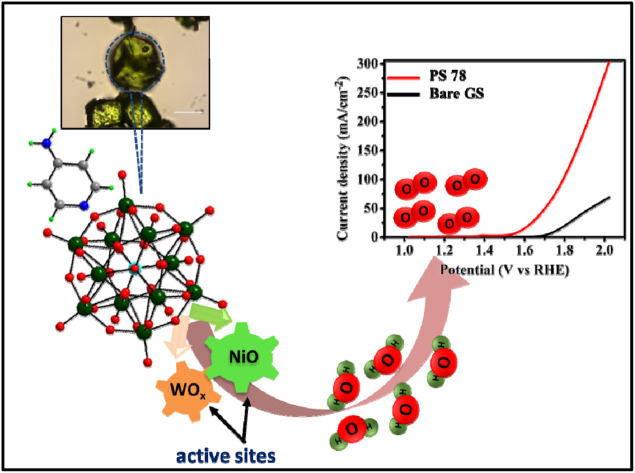

## Introduction

**Scheme 1:**
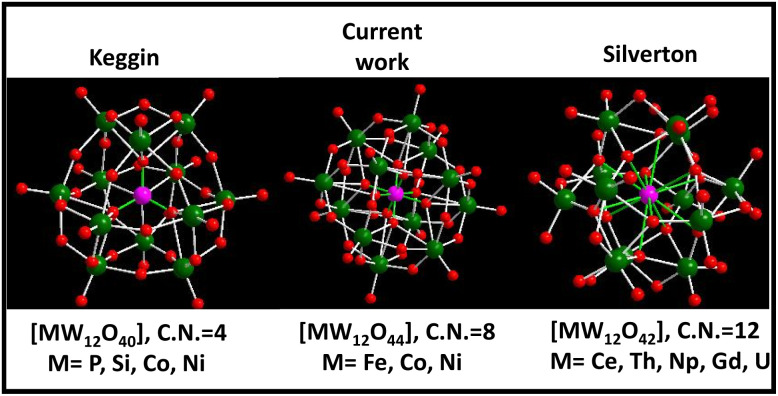
Different hetero-polyoxometalates belonging to the 1 : 12 series of POMs with various heteroatoms.

Polyoxometalates (POMs) are a well-known and fascinating family of atomically precise inorganic anionic nanoclusters made up of early transition metals (Mo, W, V *etc.*) in their maximum oxidation state.^1–3^ POMs exhibit huge structural diversity that simultaneously offers a broad range of applications particularly in the energy and catalysis fields.^4,5^ The most cutting-edge and pressing issue in POM chemistry was the discovery of the basic POM topological structure. The six fundamental structural branches of the POM family include Keggin, Dawson, Anderson, Silverton, Waugh, and Lindqvist, with Keggin and Silverton POMs being the members of the 1 : 12 hetero-polyoxometalate series. Keggin's structural variety is greater than that of Silverton, as various metals other than Mo and W can also build the Keggin cluster skeleton with an incorporated heteroatom that might be a non-metal element (*e.g.*, P, Si, B and As) or a transition metal (*e.g.*, Fe, Co and Ni) having a coordination number (CN) of 4 (Scheme 1).^1,6^ Meanwhile, the documented structure of Silverton POMs is very less, and only Mo-based Silverton POMs of [MMo_12_O_42_] with lanthanide elements (Ce and Gd) and actinide elements (Th, U, and Np) as the central heteroatom in the 12 CN state have been reported so far (Scheme 1).^7^ Thus there is a missing gap between 1 : 12 hetero-POMs of Keggin and Silverton that is of a POM structure having the central heteroatom with a coordination number of 8. There is a pressing need to fix this problem in POM chemistry. In this regard, there is only one existing report where the researchers attempted to synthesize [MW_12_O_44_]^14−^ cluster based hetero-POMs using [W_12_O_44_]^16−^ as a structure-directing precursor in a precise and constant pH environment provided by a glycine–hydrochloric acid buffer.^8^ In the present report, we have employed a general procedure that is very common in the synthesis of POM-based solids to synthesize [NiW_12_O_44_]^14−^ cluster based POMs having a central heteroatom with a CN of 8 (Scheme 1), functionalized with a 4-aminopyridine ligand. Our group is currently working on incorporating other heteroatoms (Co, Cu and Fe) in the [W_12_O_44_] cluster.

The ever-increasing emissions of harmful CO_2_ pushes us to seek out cleaner renewables to replace commonly used fossil fuels.^9^ In addition to its high conversion efficiency and gravimetric energy density, H_2_ has been touted as a suitable carbon-free energy transporter.^10^ Water electrolysis has been regarded as the primary and easiest method for scalable H_2_ synthesis to date, but the performance of anodic water oxidation, specifically, the oxygen evolution reaction, is still a major limiting issue.^11,12^

The OER has unusually slow kinetics and, in the meantime, causes structural changes in electrode materials, resulting in a drop in performance and, eventually, electrode disintegration.^13^

The best OER activity has so far been provided by ruthenium (Ru) and iridium (Ir) oxides,^14,15^ but their scarcity, high cost, and quick deactivation severely limit their practical usage.

POMs have certain unique qualities, including redox activity, oxidative resilience, molecular level structural fine tailoring, and complete inorganic framework, which have made them popular candidates in the area of water oxidation catalysis in recent years.^16–18^ Specifically, cobalt POMs (Co-POMs) have attracted major attention as WOCs,^19–24^ and nickel POMs are totally untouched in this field. Also, it's a matter of debate long back since POMs started serving as WOCs, whether POMs behave as a true catalyst or as a precursor material to produce active species for WO mainly under an electrochemically driven water oxidation process.^25^ According to certain studies, Co-POMs act as precursors to leached Co(ii) aq and later heterogeneous cobalt oxide (CoO_*x*_), which is a real, kinetically dominant WO catalyst.^25,26^ POMs in WO are preferred over the direct use of metal oxides as POM clusters and also offer more exposed active sites because of their redox nature. Also, during electro-oxidation, complete reconstruction of POMs occurs, which leads to a unique structure of the corresponding metal oxides, oxy-hydroxides and/or hydroxides as extraordinarily stable OER-active species. Liu *et al.* compared the OER activity of NiFe-LDH with and without a POM and it was found that the material with the POM has better activity than that of without the POM.^27^

In the past, scientists have mostly concentrated on the potential of Co-POMs to oxidize water; however, it is now time to focus on other metal-based POMs as well, since they may be better alternatives to conventional WOCs. Nickel POMs could be interesting candidates in this regard.^28^

Herein, a rare POM cluster [MW_12_O_44_]^14−^ based solid, (C_5_H_7_N_2_)_6_[NiW_12_O_44_] (PS-78), has been reported, whose optical microscopic images are shown in Fig. S1.† The solid has been fully characterized by different analytical techniques like single crystal X-ray diffraction, PXRD, FTIR, thermogravimetric analysis and UV-Vis spectroscopy (details have been provided in the ESI†). Furthermore, PS-78 was investigated for water oxidation properties in alkaline medium (pH = 14). The results showed that PS-78 is an efficient WOC in alkaline medium with an overpotential of 347 mV at 10 mA cm^−2^ current density.

## Results and discussion

**Fig. 1:**
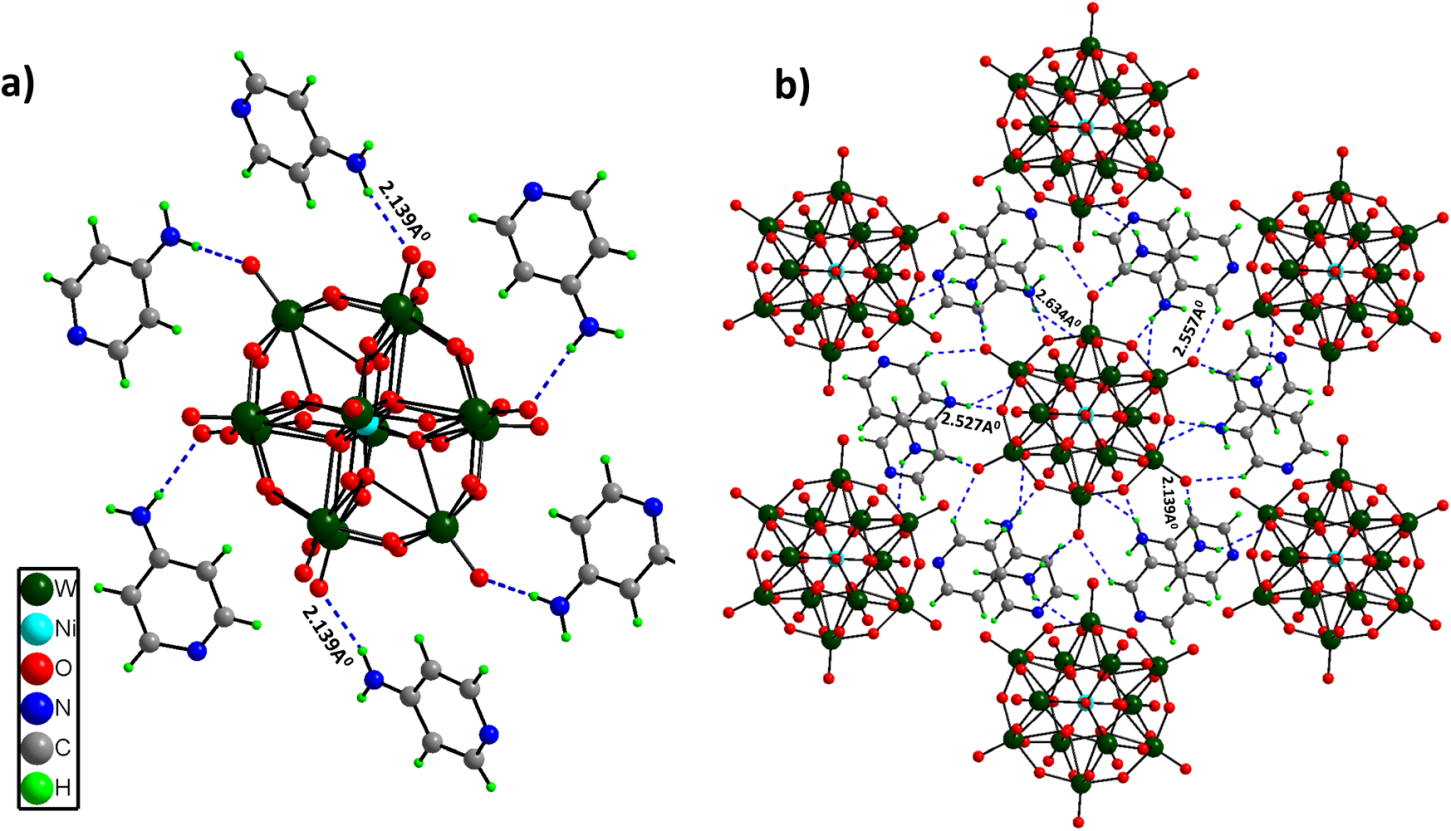
(a) Hydrogen bonding environment around the [NiW_12_O_44_]^14−^ cluster unit, and (b) three-dimensional (3D) structure of PS-78 formed by extended hydrogen bonding among 4-aminopyridine molecules and cluster's terminal oxygens.

Single Crystal X-Ray Diffraction (SCXRD) disclosed that (C_5_H_7_N_2_)_6_[NiW_12_O_44_] crystallized in a trigonal crystal system with the *R*3 space group. The structure comprises a central heteroatom Ni, which forms a hexahedron with a coordination number of 8 (NiO_8_ cube sharing edges with 12 WO_6_ octahedrons). There are three different coordination modes of oxygen atoms in this cluster: (i) 8 μ_4_-O atoms, each coordinated with the central heteroatom Ni and three W atoms; (ii) 24 μ_2_-O atoms, each coordinated with two W atoms; (iii) 12 terminal O atoms, each coordinated with one tungsten atom. The W–O bond distances can be divided into three distinct groups depending on the type of bonding. The longer W–O distances of 2.428 Å correspond to μ_4_-O (coordinated to three W atoms and one central heteroatom Ni). The medium W–O distances of 1.9 Å correspond to μ_2_-bridged W–O bonds, while the shorter distances of 1.712 Å and 1.703 Å correspond to terminal ones. Furthermore, [NiW_12_O_44_]^14−^ clusters are involved in hydrogen bonding interactions with the –NH_2_ group of 4-aminopyridine molecules (2.139–2.634 Å), leading to a 3D framework structure (Fig. 1a and b). Crystallographic information and asymmetric units (Fig. S2†) are provided in the ESI (Table S1†).

PS-78 was further characterized by different analytical techniques. The powder X-ray diffraction (PXRD) pattern confirms the phase purity and homogeneity of PS-78 (Fig. S3†). The presence of a polyoxotungstate framework in PS-78 was validated by ATR-FTIR. FTIR spectra (Fig. S4†) show the characteristic stretching vibrations (in cm^−1^) of POMs at 950 (terminal W=O), 883 (W–O–W) and 588 (Ni–O).^29^ Furthermore, the peaks of 4-aminopyridine were assigned to the frequencies (in cm^−1^) 3415, 3332 and 1651.^30^ The thermogravimetric analysis (TGA) curve (Fig. S5†) revealed two major weight losses, the first at 400 °C due to organic moiety removal and the second around 610 °C due to POM cluster destruction. After 900 °C, there was a slight weight gain, which could be attributed to the formation of nickel and tungsten oxides.^31^ The absorption properties of PS-78 were further investigated by UV-Vis spectroscopy. A ligand to metal charge transfer (LMCT) band (O π to W^VI^ t_2g_*) was observed at 260 nm (Fig. S6†). The d–d transition absorption band is considered as a fingerprint characteristic peak for doped POMs; in the case of PS-78, it was detected at 580 nm (Fig. S7†) and attributed to the nickel centered lowest energy electronic transition from HOMO t_2g_* to LUMO e_g_*.^32^

**Fig. 2:**
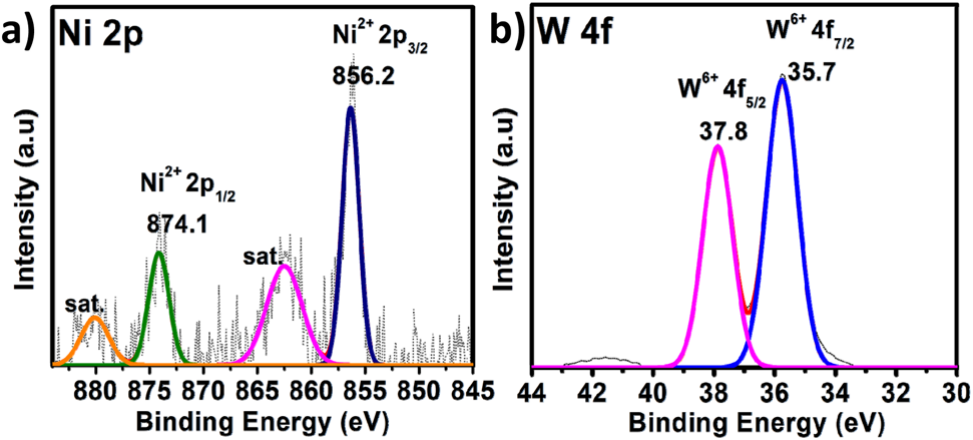
XPS of PS-78. The high-resolution spectra of (a) Ni 2p and (b) W 4f. The dotted curve demonstrates the experimental data, while the solid line displays the results of fitting.

X-ray photoelectron spectroscopy (XPS) is a significant tool for determining the elemental composition of a sample as well as the oxidation states of the elements present (survey spectrum, Fig. S8†). The oxidation state of W and the heteroatom Ni in PS-78 was characterized by XPS. The two XPS peaks located at 880 and 874.1 eV correspond to the Ni^2+^ (2p_1/2_) orbital binding energies, whereas peaks at 862.4 and 856.2 eV are assigned to the Ni^2+^ (2p_3/2_) orbital binding energies (Fig. 2a). It clearly denoted that the oxidation state of Ni was Ni^II^. Peaks at 35.8 and 35.7 eV (Fig. 2b) are assigned to W^6+^ (4f_5/2_) and W^6+^ (4f_7/2_) orbital binding energies, showing that W has the highest oxidation state, W^VI^. Furthermore, to determine the morphology of PS-78, FESEM analysis was done. FESEM images revealed that PS-78 doesn't have any particular morphology (Fig. S9†). However, the FESEM elemental mapping result supports the results of XPS and SCXRD and confirms the presence and even distribution of the elements Ni, W, C, O and N (Fig. S10†) in PS-78.

The electrochemical oxygen evolution catalytic properties of PS-78 were investigated by using a regular three electrode setup on a CHI workstation in alkaline medium, 1 M KOH (pH 14), where PS-78 (the sample prepared in ethanol, and detailed experimental details have been provided in the ESI†) drop casted on a graphitic strip (GS) acted as the working electrode while a graphitic rod served as the counter electrode and Ag/AgCl as the reference electrode.

**Fig. 3:**
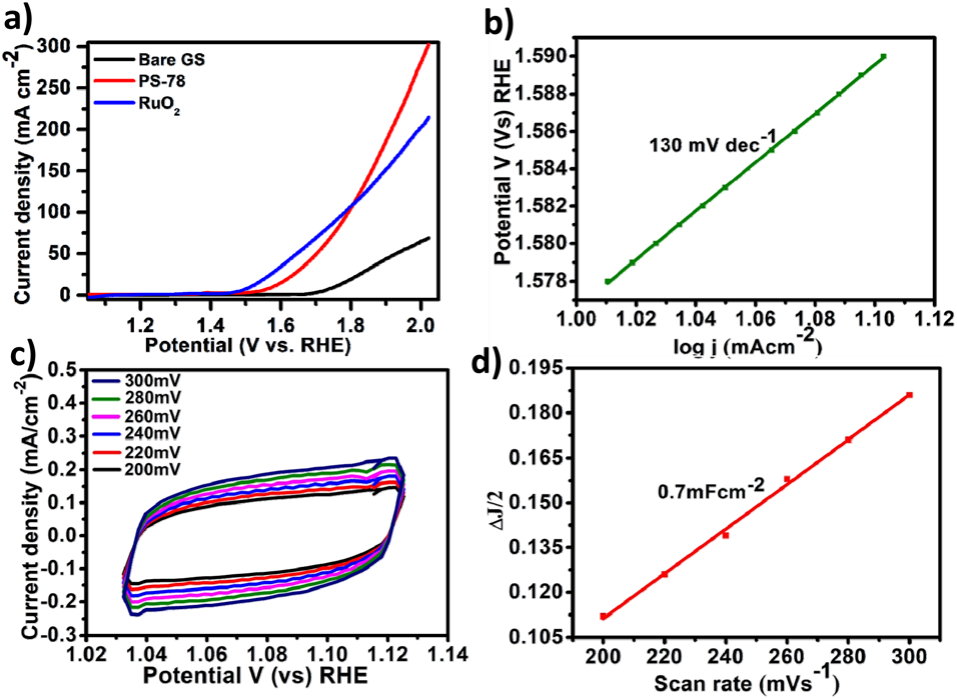
(a) Polarization curves of PS-78 and the substrate (GS) at a scan rate of 5 mV s^−1^, (b) Tafel plot of PS-78, (c) cyclic–voltammetry curves of PS-78 at different scan rates (200–300 mV s^−1^) and (d) double layer charge capacitance (*C*_dl_) of PS-78.

**Fig. 4:**
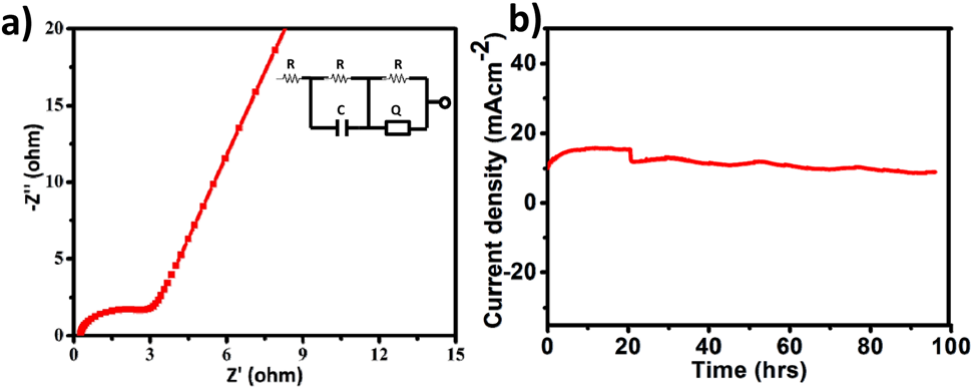
a) Electrochemical impedance spectrum of PS-78 (fitted circuit is demonstrated in the inset), and (b) chronoamperometric curve of PS-78 at *E* = 1.57 V.

All electrochemical potentials are referenced to the Reversible Hydrogen Electrode (RHE). The Linear Sweep Voltammetry (LSV) curve demonstrated the efficient OER performance of PS-78 (Fig. 3a), and PS-78 required an overpotential of 347 mV to attain a current density of 10 mA cm^−2^. The LSV curve of the substrate (bare GS) and commercially used catalyst RuO_2_ was also recorded for comparison (Fig. 3a). The Tafel slope value derived from the LSV curve was determined to be 130 mV dec^−1^ and suggested kinetically favoured OER performance (Fig. 3b). To find out the value of double layer charge capacitance, cyclic voltammograms were recorded at different scan rates (200, 220, 240, 260, 280 and 300 mV s^−1^) (Fig. 3c) and the value was obtained to be 0.7 mF cm^−2^ (Fig. 3d). Furthermore, electrochemical impedance measurements were carried out to quantify electrode kinetics and interface reactions. The lower semicircle diameter of the Nyquist plot indicates lower charge transfer resistance (*R*_ct_) and higher charge transfer (Fig. 4a). The value of *R*_ct_ for the catalyst was found to be 2.8 Ω, which suggested favorable charge transfer at the electrode–electrolyte interface. A comparison table of PS-78 with other reported POM-based electrocatalysts, in terms of activity and stability, has been provided (Table 1). It shows the long-term stability of our catalyst.

**Table 1:** Comparison of the catalytic performance and stability of reported POM-based materials

Material	Electrolyte (KOH)	*j*	*η*	Stability	Ref
	(M)	(mA cm^−2^)	(mV)	(hours)	
SiW_11_Co@ZIF-67	0.1	10	460	—	40
[Co_6.8_Ni_1.2_W_12_O_42_(OH)_4_(H_2_O)_8_]	0.1	10	340	10	41
PBA@POM	1	10	440	18	42
WS_2_/Co_1−*x*_S/N	1	10	365	—	43
ZIF-67@ POM	1	10	287	12	44
AB & PS-13 (1 : 2)	1	10	330	24	18
[Ni(2,2′-bpy)_3_]_3_[{Ni(2,2′-bpy)_2_(H_2_O)}{HCoW_12_O_40_}]_2_·3H_2_O	0.1 M phosphate buffer	10	475.6	20	28
[{Ru_4_O_4_(OH)_2_(H_2_O)_4_}(SiW_10_O_36_)_2_]^10−^	0.2 M phosphate buffer	10	550	10	45
[{FeCo_3_(OH)_3_PO_4_}_4_(SiW_9_O_34_)_4_]^28−^	0.5 M H_2_SO_4_	10	385	24	46
**(C**_**5**_ **H**_**7**_ **N**_**2**_ **)[NiW**_**12**_ **O**_**44**_ **]**	**1 M KOH**	**10**	**347**	**96**	**Present work**

The stability and long-term performance are the most crucial factors for any catalyst to be employed in practical applications. The chronoamperometry curve of PS-78 at a current density of 10 mA cm^−2^ (*E* = 1.57 mV) was utilized to assess the performance stability of the catalyst, PS-78 (Fig. 4b). PS-78 can hold a current of 10 mA cm^−2^ up to 96 hours, implying that the catalyst can provide continuous oxygen evolution under experimental conditions for up to 96 hours, and it suggests sustainable and long-term OER performance of PS-78.

**Fig. 5:**
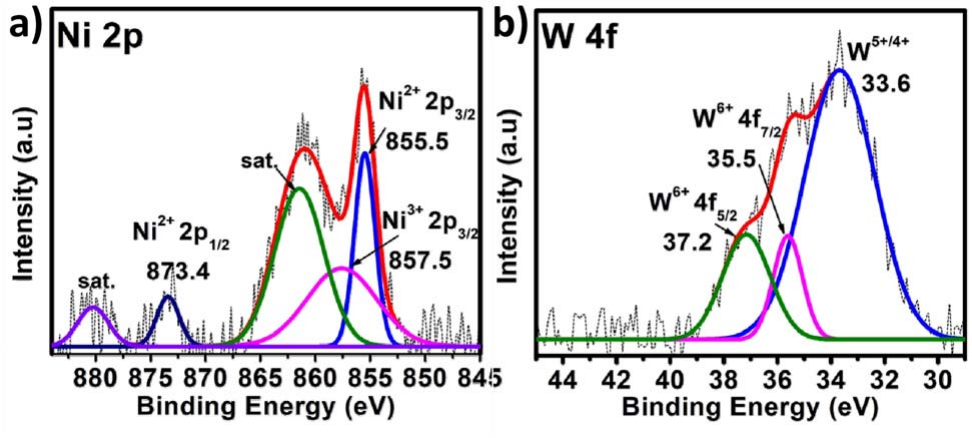
XPS of PS-78 post OER. The high resolution spectra of (a) Ni 2p and (b) W 4f. The dotted curve demonstrates the experimental data, while the solid line displays the results of fitting.

In the case of polyoxometalates, the mechanistic pathway of the oxygen evolution reaction is a debatable issue. Previous studies have demonstrated that Co-based POMs, like [Co_4_(H_2_O)_2_(PW_9_O_34_)_2_]^10−^, can act as an efficient OER catalyst, although real active sites in these materials are still being investigated.^26^ Some researchers have shown that during electrocatalysis, some *in situ* conversion of POMs occurs, and these species there after serve as true active sites for oxygen evolution, giving rise to the term “pre-catalyst” for the subjected POM. In the current study, the chronoamperometry curve revealed that there is a slight increase in current density between 0 and 20 hours, but after 20 hours, it becomes constant, and this consistency lasts for 96 hours. It appears that some *in situ* alteration occurs within the catalyst in the early hours, followed by the formation of stable and efficient species. To further investigate the active sites for the OER, post-OER characterization of PS-78 was performed. PXRD analysis revealed the presence of WO_*x*_ (*x* = 1, 2), NiO, Ni(OH)_2_ and NiOOH in the material^33,34^ (Fig. S11†) and further this result was backed up by XPS analysis as well. The XPS survey spectrum of PS-78 after the OER validates the presence of W, Ni, C, O and N elements (Fig. S12†), as presented in the as-synthesized PS-78. The high resolution XPS spectra of W 4f depicted three peaks located at 33.6, 35.5 and 37.2 eV corresponding to W^+5/+4^ 4f, W^+6^ 4f_7/2_ and W^+6^ 4f_5/2_ respectively (Fig. 5b), which confirms the presence of WO_*x*_ (*x* = 1, 2) in the material (PS-78) after the OER.^35^ The two XPS peaks located at 880.2 and 873.4 eV are assigned to the Ni^2+^(2p_1/2_) orbital binding energies, whereas the peaks at 855.5 and 857.5 eV are assigned to the Ni^2+^ (2p_3/2_) and Ni^3+^ (2p_3/2_) orbital binding energies (Fig. 5a), respectively.^36^ The existence of Ni^3+^ indicates that NiOOH and Ni(OH)_2_ are present.^36*b*,37^ In light of the post-OER investigation, it can be concluded that during the electrocatalysis process, PS-78 undergoes *in situ* conversion, resulting in the formation of NiO and WO_*x*_ (*x* = 1,2) species, which serve as real active sites for the OER, exhibiting efficient and long-term oxygen evolution.^38,39^

## Conclusions

A rare polyoxometalate cluster [NiW_12_O_44_]^14−^ based solid (PS-78) has been synthesized and characterized by various techniques. This cluster, having an 8-coordinated central heteroatom Ni, bridges the gap between Keggin and Silverton clusters of the 1 : 12 POM family. PS-78 was further investigated for electrocatalytic oxygen evolution. It demonstrates sustained oxygen evolution with an overpotential of 347 mV at 10 mA cm^−2^ current density with a stability of at least 96 hours. Post-catalytic analysis revealed PS-78 as a pre-catalyst and demonstrated the presence of Ni(OH)_2_, NiOOH, and WO_*x*_ species during electrocatalysis, which serve as real active sites for the OER. This research opens up a new path for investigating POM-based materials in oxygen evolution reactions and identifying real active sites.

## Conflicts of interest

There are no conflicts to declare.

## Supplementary Material

NA-004-D2NA00646D-s001

NA-004-D2NA00646D-s002
