# Supplementary material for: A rare polyoxometalate cluster [NiW12O44]14− based solid as a pre-catalyst for efficient and long-term oxygen evolution
Source: Nanoscale Adv. 2022 Oct 13;4(23):5015–20. doi: 10.1039/d2na00646d (PMC9680933; doi:10.1039/d2na00646d)
Supplement: NA-004-D2NA00646D-s001 [file NA-004-D2NA00646D-s001.pdf]

Electronic Supplementary Material (ESI) for Nanoscale Advances.
This journal is © The Royal Society of Chemistry 2022

Supporting Information

A rare polyoxometalate cluster $[NiW_{12}O_{44}]^{14-}$ based solid as a pre-catalyst for efficient and long-term oxygen evolution

Parul Sood,[†a] Arti Joshi[†a] and Monika Singh[*a]

[a] Energy and Environment Unit
Institution: Institute of Nano Science and Technology
Address: Knowledge City, Sector-81, Mohali
E-mail: monika@inst.ac.in

Table of Contents

1. Experimental section
2. Table S1: Crystal and Structure Refinement Data for **PS-78**.
3. Figure S1. Microscopic images of **PS-78**.
4. Figure S2. Asymmetric unit of **PS-78**.
5. Figure S3. Simulated and experimental PXRD patterns of **PS-78**.
6. Figure S4. FTIR spectrum of **PS-78**.
7. Figure S5. Thermogravimetric analysis (TGA) curve of **PS-78**.
8. Figure S6. UV-vis spectrum of **PS-78**.
9. Figure S7. UV-vis diffuse reflectance (DRS) spectrum of **PS-78**.
10. Figure S8. XPS survey spectrum of **PS-78**.
11. Figure S9. FESEM images of **PS-78**.
12. Figure S10. FESEM elemental mapping of **PS-78**.
13. Figure S11. PXRD pattern of **PS-78** after OER.
14. Figure S12. XPS survey spectrum of **PS-78** after OER.

1. Experimental Section

All the reagents and solvents used were purchased of the highest grade available from commercial sources and were used without any extra purification.

1.1. Synthesis of $(C_5H_7N_2)_6[NiW_{12}O_{44}]$ (PS-78): Sodium tungstate dihydrate (1.30mmol), Nickel chloride hexahydrate (0.21mmol), 4-aminopyridine (0.48mmol), phosphoric acid (0.5mL) were added to 20mL distilled water. The pH of the solution was maintained at 2.7 with 1M NaOH. The mixture was stirred for 30 minutes and transferred to 25mL Teflon and heated for 72 hours at 160^0C. After that, the reaction was allowed to cool at room temperature. Green colored crystals were observed and collected from the walls of Teflon.

1.2. Single Crystal X-ray Diffraction: Single crystal X-ray data were collected on an Agilent Super Nova diffractometer, equipped with a multilayer optics monochromated dual source (Cu and Mo) and an Eos CCD detector, using Cu-Ka radiation (1.54184 Å) at 150 k. Data acquisition, reduction and absorption correction were performed by using CrysAlisPRO.[1] The structure was solved with ShelXS[2] program using direct methods and refined on F 2 by full matrix least-squares techniques with ShelXL[2] through the Olex 2 (v.1.2) program package.[3] Anisotropic displacement parameters were applied for all the atoms, except for hydrogen atoms. The hydrogen atoms were placed in calculated positions and refined as riding atoms using isotropic displacement parameters. Images were created with the Diamond program.[4] The crystal and structure refinement data are summarized in Table S1.

1.3. Physical Methods: Powder X-Ray Diffraction (PXRD) studies were carried out on a Bruker D8 – Advance Eco X-ray Diffractometer with Cu-Kα radiation (1.5418 Å). The morphological analysis of the catalyst was done by using a field emission scanning electron microscope (FESEM) from JEOL (JSM-7600F) provided with energy-dispersive X-ray diffractometer (Bruker). Valence states of the elements were found by X-Ray Photoelectron Spectroscopy (XPS) spectrometer (K-Alpha 1063).

1.4. Electrochemical Study: All electrochemical measurements were executed on a CHI electrochemical workstation. In the conventional three electrode setup graphite electrode is used as a counter electrode, a saturated Ag/AgCl electrode as a reference electrode. The ink for the coating of graphitic strip electrode was prepared by using 5 mg of the catalyst in 500 μL of ethanol containing 20 μL of 5 wt% Nafion. The mixture was sonicated for 120 min and

the homogeneous suspension was prepared. Out of this solution only 80 μL was coated on graphitic strip electrode and dried in vacuum. All the polarization data were collected at the scan rate of 10 mV s^{-1} in 1 M KOH. The equation used for the conversion to reversible hydrogen electrode is $E_{RHE} = E_{Ag/AgCl} + 0.197 + 0.059*pH$. The determination of Tafel slope was done by fitting the linear region of the Tafel plot to the Tafel equation ($\eta = b \log(j) + a$) by again plotting the polarization curve. The double layer capacitance was calculated for the determination of electrochemically active surface area (ECSA) analysis by the CV scans in a non-Faradaic potential range of as-prepared catalysts electrodes in 1 M KOH at a scan rates 200, 220, 240, 260, 280 and 300mV/s. Half of the differences in current density variation ($\Delta J = (J_{anodic}-J_{cathodic})/2$) at a potential of 1.08 V vs RHE plotted against scan rate fitted to a linear regression that allows the determination of double layer capacitance (C_{dl}). The faradic efficiency was determined by calculating the ratio of the experimental and theoretical oxygen evolution.

Table S1: Crystal and Structure Refinement Data for PS-78.

**Parameter**	$(C_5H_7N_2)_6[NiW_{12}O_{44}]$
Formula	C30 H36N12NiO44W12
Formula weight, g	3533.48
T (K)	150.0(2)
Wavelength (Å)	1.54184
Crystal system	Trigonal
Space Group	R-3(148)
a (Å)	21.9309(14)
b (Å)	21.9309(14)
c (Å)	10.3647(7)
α (°)	90
β (°)	90
γ (°)	120
V ($Å^3$)	4317.2(6)
Z	3
dcalc (gcm^{-3})	4.077
$\mu CuK\alpha$, (cm^{-1})	2.303
R1($I>2\sigma I$)	0.0711
WR2(all)	0.1998
CCDC/CSD No.	2132841

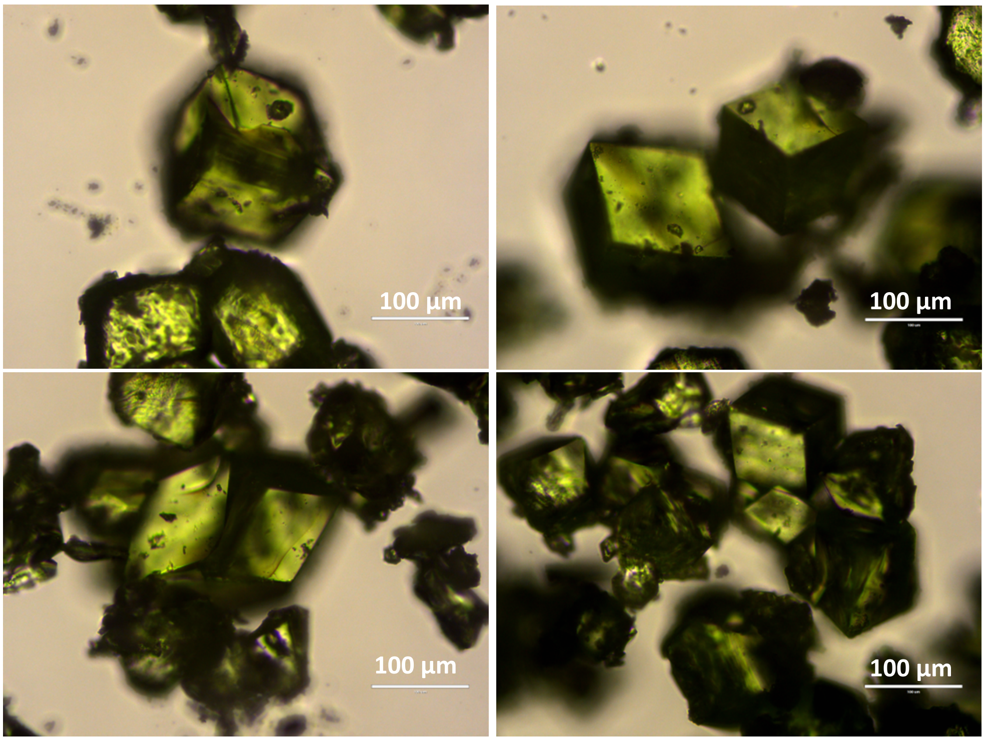

Figure S1. Optical microscopic images of **PS-78**.

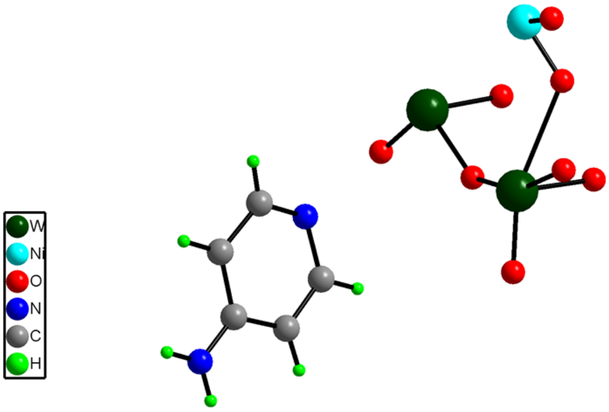

Figure S2. Asymmetric unit of **PS-78**.

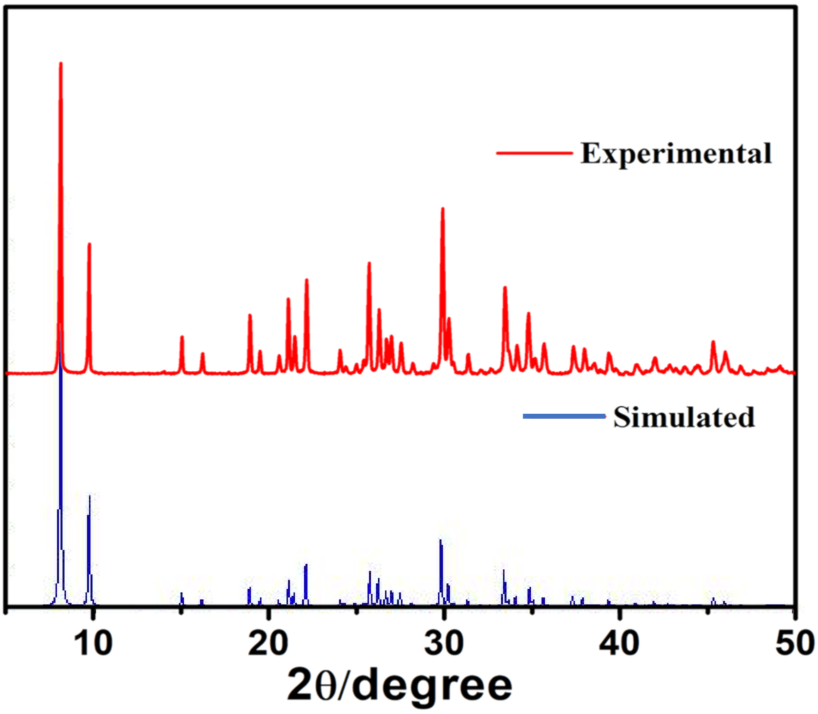

Figure S3. Simulated and experimental PXRD patterns of **PS-78**.

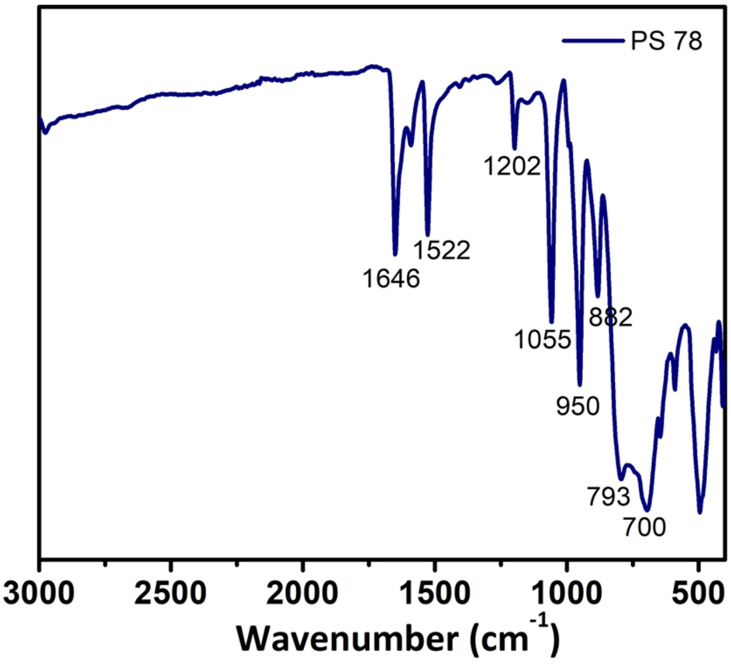

Figure S4. FTIR spectrum of **PS-78**.

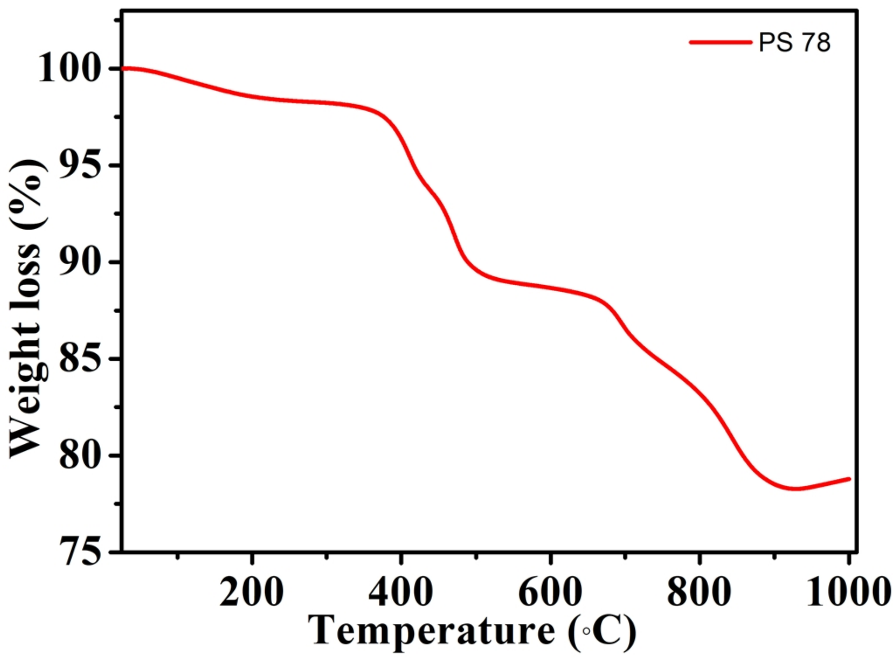

Figure S5. Thermogravimetric analysis (TGA) curve of **PS-78**.

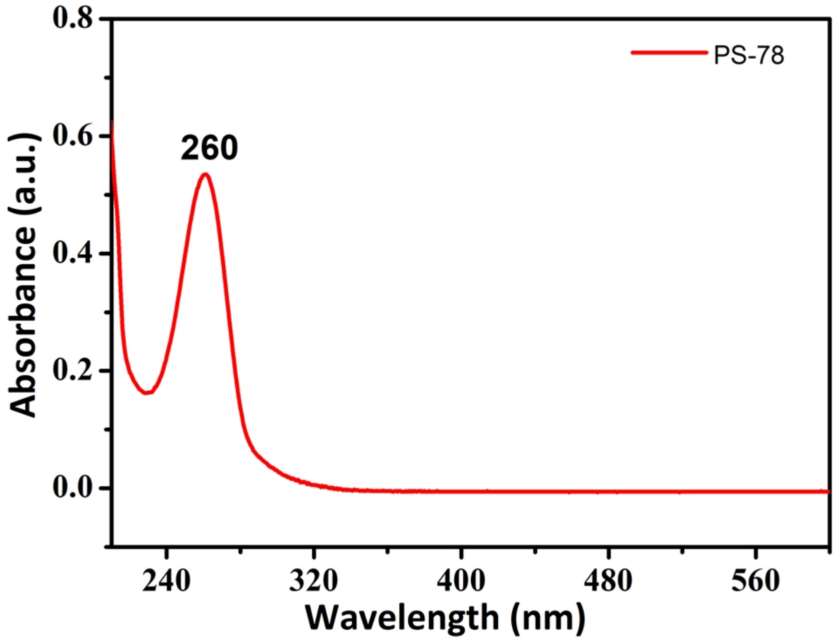

Figure S6. UV-vis spectrum of **PS-78**.

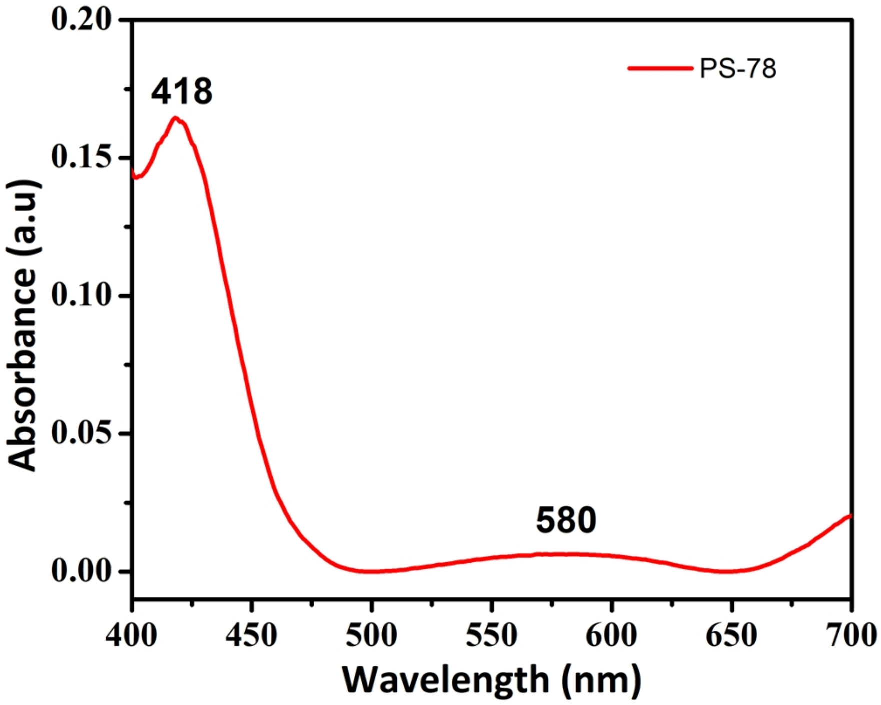

Figure S7. UV-vis diffuse reflectance (DRS) spectrum of **PS-78**.

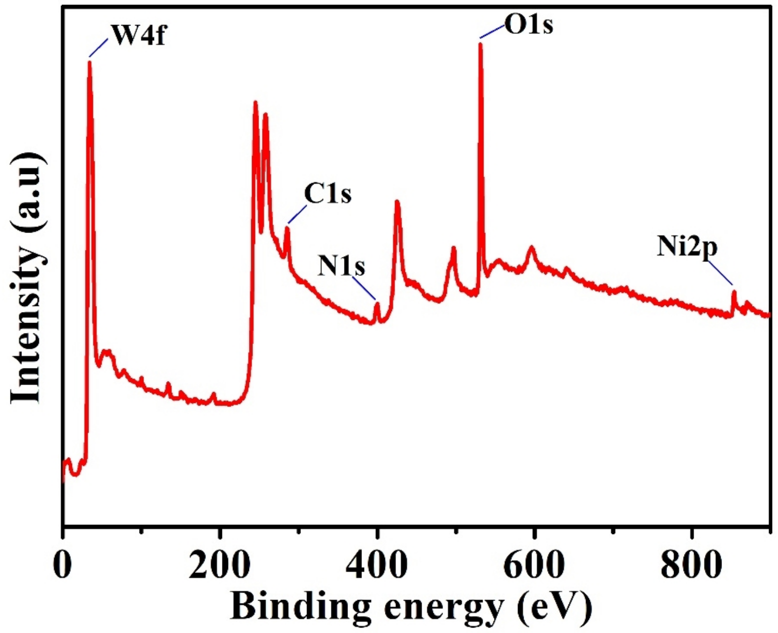

Figure S8. XPS survey spectrum of **PS-78**.

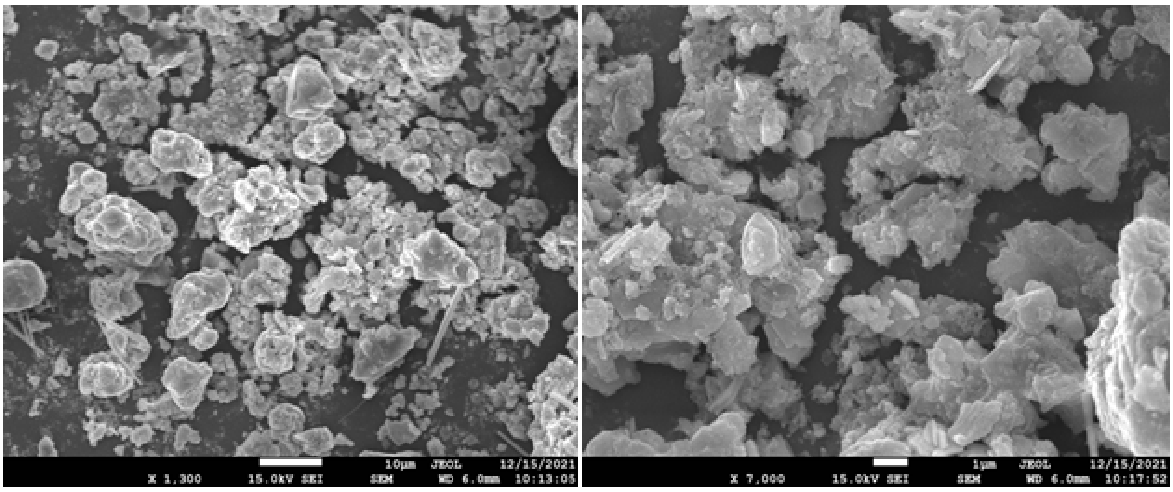

Figure S9. FESEM images of **PS-78**.

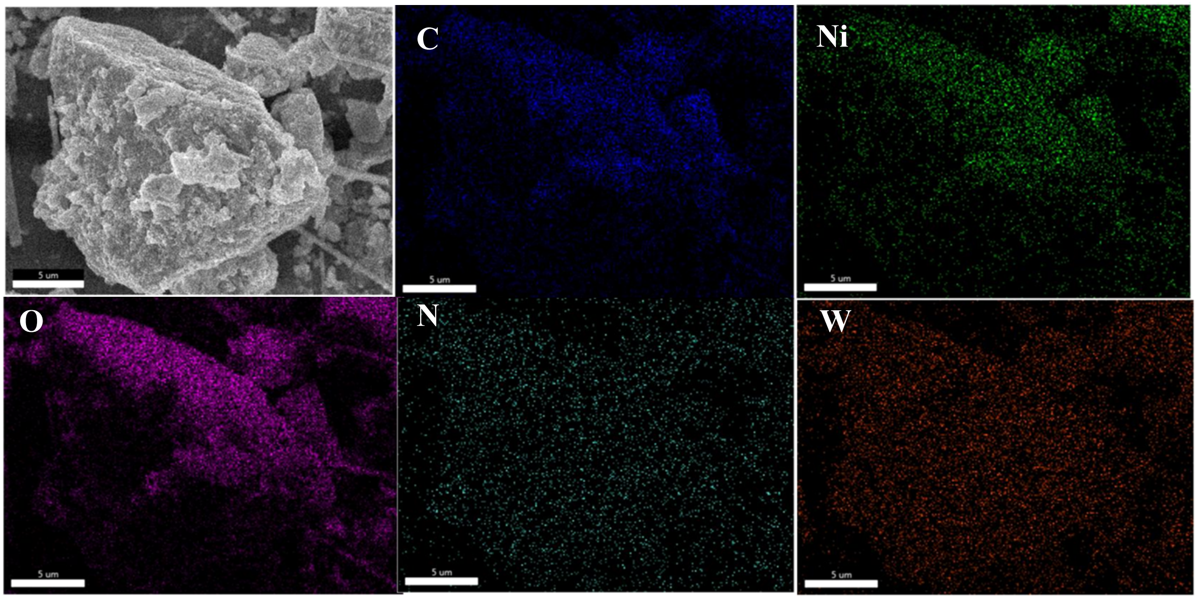

Figure S10. FESEM elemental mapping of **PS-78**.

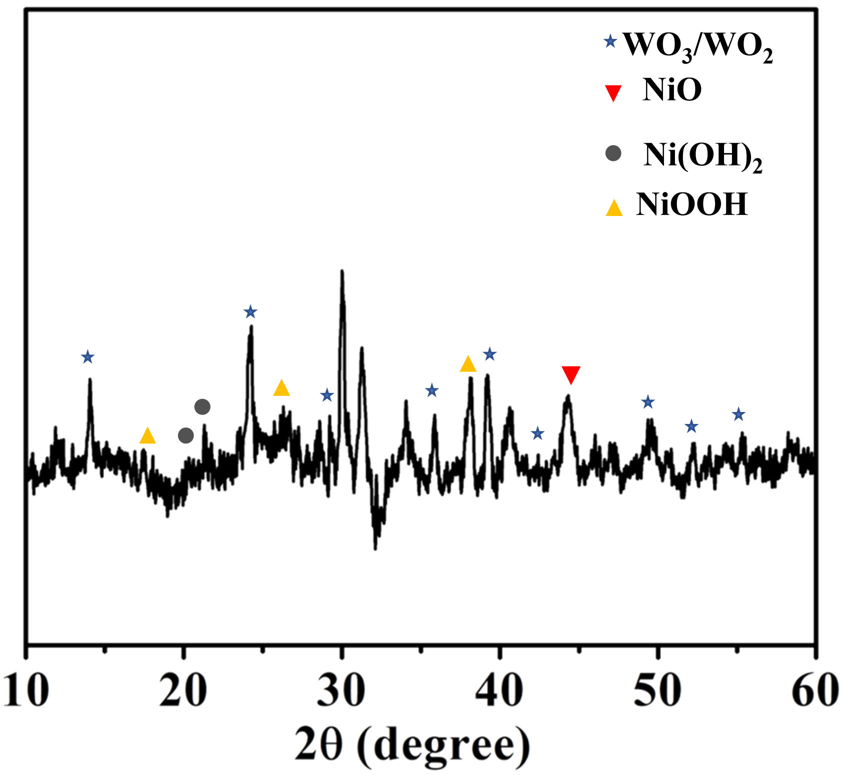

Figure S11. PXRD pattern of **PS-78** after OER.

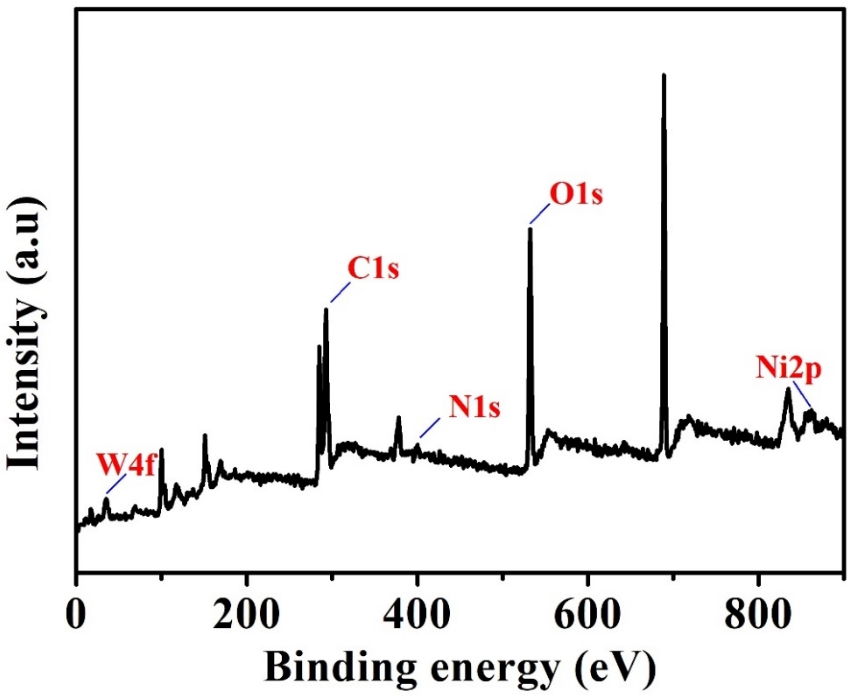

Figure S12. XPS survey spectrum of **PS-78** after OER.

References:

1. Bruker Analytical X-ray Systems, SMART: Bruker Molecular Analysis Research Tool, Version 5.618; Bruker AXS: Madison, WI, 2000.

2. Bruker Analytical X-ray Systems, SAINT-NT, Version 6.04; Bruker AXS: Madison, WI, 2001.

3. Bruker Analytical X-ray Systems, SHELXTL-NT, Version 6.10; Bruker AXS: Madison WI 2000.

4. Klaus, B. DIAMOND, version 1.2c; University of Bonn: Germany, 1999.
